# UMBRELLA protocol: systematic reviews of multivariable biomarker prognostic models developed to predict clinical outcomes in patients with heart failure

**DOI:** 10.1186/s41512-020-00081-4

**Published:** 2020-08-26

**Authors:** Maria D. L. A. Vazquez-Montes, Thomas P. A. Debray, Kathryn S. Taylor, Benjamin Speich, Nicholas Jones, Gary S. Collins, F. D. R. Richard Hobbs, Emmanuella Magriplis, Hugo Maruri-Aguilar, Karel G. M. Moons, John Parissis, Rafael Perera, Nia Roberts, Clare J. Taylor, Nikolaos P. E. Kadoglou, Marialena Trivella, on behalf of the proBHF group

**Affiliations:** 1 Nuffield Department of Primary Care Health Sciences, Radcliffe Observatory Quarter, University of Oxford, Woodstock Road, Oxford, OX2 6GG UK; 2 Centre for Statistics in Medicine, Nuffield Department of Orthopaedics, Rheumatology and Musculoskeletal Sciences, Botnar Research Centre, University of Oxford, Windmill Road, Oxford, OX3 7LD UK; 3 Julius Center for Health Sciences and Primary Care, University Medical Center (UMC), Utrecht University, Heidelberglaan 100, 3584 CX Utrecht, The Netherlands; 4 Basel Institute for Clinical Epidemiology and Biostatistics, Department of Clinical Research, University Hospital Basel, University of Basel, Basel, Switzerland; 5 National Institute for Health Research Oxford Biomedical Research Centre, John Radcliffe Hospital, Oxford, UK; 6 Department of Food Science and Nutrition, Agricultural University of Athens, Iera Odos, 75 Athens, Greece; 7 School of Mathematical Sciences, Queen Mary University of London, E1 4NS, London, UK; 8 Second Department of Cardiology, Attikon University Hospital, National and Kapodistrian University of Athens, Athens, Greece; 9 Bodleian Health Care Libraries, University of Oxford, Oxford, UK

**Keywords:** Acute heart failure, Decompensated heart failure, Chronic heart failure, Biomarkers, Prediction rule, Risk score, Prediction accuracy, CHARMS, PROBAST, Prognosis

## Abstract

**Background:**

Heart failure (HF) is a chronic and common condition with a rising prevalence, especially in the elderly. Morbidity and mortality rates in people with HF are similar to those with common forms of cancer. Clinical guidelines highlight the need for more detailed prognostic information to optimise treatment and care planning for people with HF. Besides proven prognostic biomarkers and numerous newly developed prognostic models for HF clinical outcomes, no risk stratification models have been adequately established. Through a number of linked systematic reviews, we aim to assess the quality of the existing models with biomarkers in HF and summarise the evidence they present.

**Methods:**

We will search MEDLINE, EMBASE, Web of Science Core Collection, and the prognostic studies database maintained by the Cochrane Prognosis Methods Group combining sensitive published search filters, with no language restriction, from 1990 onwards. Independent pairs of reviewers will screen and extract data. Eligible studies will be those developing, validating, or updating any prognostic model with biomarkers for clinical outcomes in adults with any type of HF. Data will be extracted using a piloted form that combines published good practice guidelines for critical appraisal, data extraction, and risk of bias assessment of prediction modelling studies. Missing information on predictive performance measures will be sought by contacting authors or estimated from available information when possible. If sufficient high quality and homogeneous data are available, we will meta-analyse the predictive performance of identified models. Sources of between-study heterogeneity will be explored through meta-regression using pre-defined study-level covariates. Results will be reported narratively if study quality is deemed to be low or if the between-study heterogeneity is high. Sensitivity analyses for risk of bias impact will be performed.

**Discussion:**

This project aims to appraise and summarise the methodological conduct and predictive performance of existing clinically homogeneous HF prognostic models in separate systematic reviews.

Registration: PROSPERO registration number CRD42019086990

## Background

This in an umbrella protocol covering a number of systematic reviews in the area of heart failure. Clinically homogenous data will be considered together in each systematic review, while the clinical outcomes listed below will be explored where possible in all the resulting reviews.

### Heart failure epidemiology

Heart failure (HF) is a complex disease related to a structural and/or functional cardiac abnormality which impairs the ability of the heart to function as an efficient blood pump. With a rising prevalence (currently estimated between 6 and 10% in people older than 65 years) primarily due to population ageing, HF is now a major public health problem affecting approximately 26 million people worldwide [1–3]. In 2012, it was estimated that HF is responsible for health expenditures as high as 31 billion US$ worldwide and costs seem to be rising [4].

People with HF may be categorised in terms of symptom stability. Acute HF (AHF) refers to either onset of symptoms in people with previously unknown HF (de novo HF) or to a recent decompensation of previously stable HF symptoms, in contrast to people with chronic HF (CHF) who have had an extended period of symptom stability. CHF may also be categorised according to the individual’s left ventricular ejection fraction (LVEF) into: preserved ejection fraction (HFpEF) if LVEF≥50%, mid-range ejection fraction (HFmrEF) if LVEF ranges between 40 and 49%, and reduced ejection fraction (HFrEF) if LVEF<40% [5].

People with HF may require hospitalisations and frequent re-admissions [6]. In the United Kingdom, CHF accounts for 2% of all National Health Service (NHS) hospital admissions and costs approximately 2% of the annual NHS budget [7]. People diagnosed with AHF typically have a poor prognosis, with a mortality rate of around 40% within a year of diagnosis [8], whereas for CHF patients, this rate is around 20% [5, 9]. Overall, 5-year survival rates for people with advanced HF are worse than for people with common forms of cancer like breast or prostate cancer [10].

The National Institute for Health and Care Excellence (NICE) guidelines [11] recommend the following actions as some of the key factors for improving quality of life, reduce hospitalisation frequency, and increase survival: early diagnosis, accurate assessment, providing prompt prognoses, and timely intervention [8, 12–14]. Current pharmacological and non-pharmacological interventions have been shown to increase the life expectancy of HF patients and reduce the number of related hospitalisations [11, 15]. However, there has not been conclusive evidence supporting an improvement in hospitalisation rates in HFpEF [16]. Also, it has been demonstrated from clinical registry data that after each episode of acute HF, the prognosis of HF patients worsens, the risk of re-hospitalisation increases, and patients often do not receive optimised treatment (recommended care path, medication type, and dose for the individual’s clinical characteristics) during or after each acute HF episode [17, 18]. This is partly attributed to poor adherence to current guidelines [19] and a lack of widely accepted risk stratification models for HF [11, 15, 20].

### Prognostic factors and models

Prognostic factors are clinical or biological patient characteristics that are related to certain disease outcomes. Biomarkers, which we define as biological factors measured in blood samples, may also serve as prognostic factors. In HF, the prognostic abilities of many biomarkers [21–25] have been investigated [22, 26]. Sometimes, multiple factors are combined into a prognostic model. As HF treatment decisions are generally based on a combination of symptoms and laboratory findings, by including the prognostic potential of multiple biomarkers, we may be able to better differentiate between individuals’ needs and assist clinicians in offering maximum optimal HF treatment.

Prognostic models are commonly developed in individuals with a certain diagnosis (e.g. HF) to estimate their absolute risk of future disease outcomes [27]. They are mathematical expressions that combine multiple prognostic factors and can be used to guide treatment. A well-known example of a HF prognostic model is the Seattle Heart Failure Model (SHFM), which predicts 1-, 2-, and 3-year survival using readily available clinical, therapy, and laboratory data [28]. Another example is the Meta-Analysis Global Group in Chronic Heart Failure Risk (MAGGIC) score which predicts 3-year survival based on similar factors to those in the SHFM [29].

### Potential health outcomes

The use of prognostic models in disease management has several potential benefits [30]. For instance, model predictions can be used to inform important advanced care planning discussions with patients and their families, allowing treatment decisions to be individualised. Although some prognostic models focus on patient characteristics that are common or easy to obtain (e.g. age, gender, blood pressure levels), several studies have suggested that biomarkers such as adrenomedullin [21], high-sensitive cardiac troponin T (hs-cTnT) [22], cardiac troponin [23], soluble suppression of tumorigenicity-2 (sST2) [24], and galectin-3 [25] substantially improve their predictive performance. For this reason, prognostic models that require information on biomarkers are increasingly common in predicting clinical HF outcomes such as mortality, re-hospitalization, or advanced treatment (e.g. transplantation).

Although prognostic models are ideally developed using data from large prospective cohort studies, in practice, they are frequently derived using other available data sources such as randomised trials or databases with electronic health care records. As a result, published prognostic model studies may have limited generalisability or suffer from reduced data quality. Thus, before being introduced into clinical practice, it is essential that the predictive performance of these models is rigorously assessed in new samples (preferably from new settings) other that the one used for the model development. This requires assessment of the model’s calibration, discrimination, and impact on external validation studies [28].

### Why this work is important

Since the exploration of biomarkers became the norm first in the diagnosis and later in the prognosis of HF, there has been hundreds of prognostic models have been developed for HF. Ouwerkerk et al. in 2014 [31] summarised 117 models, while more recently Di Tanna et al. [32] identified a further 58 models published in a 5-year interval (2013 to 2018). Despite extensive work in the area, evidence on the validity and impact of these biomarker-based prognostic models on the clinical setting is lacking. Earlier systematic reviews [31, 33–35], while comprehensive in the inclusion of available models, were conducted before recent methodological advances in assessing [36], synthesising [37–39], and reporting [40, 41] prognostic models. More recent works while using up to date methodology, they have either restricted the models’ publication date to a period of 5 years [32] or chose to present a discussion paper (rather than a systematic review) on selected models [42].

Concerns about bias was common to most previously published works, as was the reported inconsistent model performance in predicting mortality. In particular, existing HF models greatly differ in quality, target population, and measured outcomes. In addition, the predictive performance of these models is rarely assessed in new settings (especially calibration) [43]. Policy makers such as NICE and the European Society of Cardiology (ESC) have therefore been reluctant to recommend the use of any prognostic model in clinical guidelines [1]. However, it is possible that refraining from using any prognostic model to guide clinical practice can lead to suboptimal treatment decisions, and potentially even be worse than basing these decisions on an inaccurate prediction model. As a first step to resolve this conundrum, we propose to perform comprehensive reviews to identify prognostic models with biomarkers for clinical outcomes in adults with all types of HF and validations thereof, assess their methodological quality, and summarise their characteristics and predictive performance. The availability of novel prognostic methodology gives us the opportunity to re-evaluate the entire body of HF prognostic modelling literature, without restrictions on HF type, year of model publication, outcome assessed, or biomarkers explored.

## Methods

The protocol is registered in PROSPERO (CRD42019086990) and follows the Preferred Reporting Items for Systematic Reviews and Meta-Analyses Protocols (PRISMA-P) 2015 statement [44] [see Additional file 3].

### Aims and objectives

This project aims to (a) identify, describe, and appraise all developed prognostic models in HF involving at least one biomarker, as well as any subsequent validation studies and to (b) summarise available data in a meta-analysis to assess each models’ predictive performance. To achieve these aims, we will conduct a number of systematic reviews to identify studies where a prognostic model has been developed and/or validated (either internally or externally), with or without any updating, according to the PICOTS items described in Table 1. The outcomes of all systematic reviews planned, along with eligibility criteria for studies and population are also listed in Table 1.

**Table 1:** PICOTS

Population	Human adult patients aged 18 or older, diagnosed with any type of HF.
Intervention (Model)	Multivariable models (i.e. models that contain two or more variables) for predicting any of the HF clinical outcomes listed below, or a combination of them, which considers, and possibly contains, prognostic factors, particularly biomarker concentrations, measured at baseline, on admission, or at discharge, or percentage change during hospitalization. The purpose of the model must be to yield absolute risk probabilities for individual patients. The biomarkers do not need to be part of the final model but considered as candidate predictors.
Outcomes	a) Mortality (either all-cause mortality, sudden cardiac death, or death from progressive pump failure); b) HF-related hospitalisation; c) need for cardiac transplantation; d) mechanical assist device implantation, independent of other present co-morbidities; and e) major adverse cardiovascular events (MACE) such as non-fatal stroke, non-fatal myocardial infarction, and cardiovascular death. Any composite of these outcomes will also be considered
Timing	No constraint will be imposed on the prediction horizon as this can vary according to the outcome predicted by each particular model. For instance, mortality could be predicted at 1, 2 or 3 years whereas re-hospitalisation could be predicted at 7 days, 1 months, or 6 months. The timing of predictor measurements could be at diagnosis of HF, discharge after a HF-related hospitalisation, or start of study recruitment.
Setting	Any setting relevant for the care of people with HF (e.g. primary care, hospital care, including emergency departments, cardiological departments, general medicine departments, intensive care units, or coronary care units).

We will summarise data only from prognostic models that predict either single or composite outcomes made up from two or more of the HF clinical outcomes stated in Table 1. Following standard systematic review meta-analysis will be attempted only in subsets of models with similar PICOTS and analysis methods. If meta-analysis is not possible results will be presented as a narrative.

### Inclusion and exclusion criteria

Table 2 lists the inclusion and exclusion criteria, separately for the type of studies and the target population.

**Table 2:** Eligibility criteria

Criteria	Type of studies	Target population
Inclusion	We will include only primary clinical studies in HF with clinical models and/or outcomes that present: - Prognostic model development, adjustment or updating with or without external validation. The model’s discrimination and/or calibration must have been reported. - External model validation. The model’s discrimination and/or calibration must have been reported. The source of data could be medical records, existing RCT data, or large clinical databases.	Adult patients aged 18 or older, diagnosed with any type of HF (i.e. ischaemic, non-ischaemic, chronic, acute, or decompensated). Patients with both reduced and preserved ejection fraction HF are eligible for inclusion. Those patients may or may not have already received optimum medical therapy (OMT), including medications and implantable devices (e.g. implantable cardioverter defibrillator (ICD) and cardiac resynchronization therapy (CRT) devices).
Exclusion	- Studies using exclusively assay analyses. - Studies published only as abstracts or clinical trials reporting no prognostic modelling on HF patients. - Studies developing models with the sole intention of evaluating the independent or adjusted association of a factor (even if this is a biomarker) with the outcome and not to predict individual probabilities. - Studies that explore the prognostic effect of treatment (eg. medication regimes, device implantation, etc.) - Systematic reviews, unless authors use a review to form a data repository for developing a prognostic model.. Their citation list will be explored for further inclusion of primary studies potentially missed by our sensitive search. - Literature reviews. - Case studies. - Diagnostic studies. - Studies focusing on economic evaluations of HF care.	- Patients who are recipients or already registered candidates for transplantation, left (LVAD), or biventricular (BiVAD) assist devices as their HF status will be significantly altered by this intervention. - Patients with advanced/end stage HF (e.g. NYHA IV) and those receiving end of life or palliative care, or who also suffer from HF as a comorbidity will not be considered because of their already established poor prognosis. - Patients with HF due to congenital conditions, and secondary to reversible causes (such as valvular disease, pregnancy and peripartum, infection, major surgery, pre-revascularisation, intensive care conditions). - Patients with concomitant disease which predominantly affects prognosis, such as cancer and neurological disorders.

### Information sources

We will search the following databases from 1990 onwards, as the biomarkers’ assays were first conducted in the 1990s, with no language restriction to reduce potential bias: MEDLINE (OvidSP); EMBASE (OvidSP); Science Citation Index & Conference Proceedings Citation Index—Web of Science Core Collection (Wok); and Database of prognostic studies maintained by the Cochrane Prognosis Methods Group (PMG). We will screen the reference lists of the included studies, relevant review articles, and practice guidelines. Authors of relevant studies, study groups, experts and investigators known to be active in the field will be contacted for unpublished material or further information on ongoing studies.

### Search strategy

We will aim for broad literature searches by targeting studies that focus on investigating prognosis in HF patients, and hence will combine published search filters for a sensitive search strategy [45]. Additional file 1 presents the search strategy. Searches will be carried out by a health information specialist (NR).

### Study records

#### Data management

Screening will be performed using Covidence [46] and selected articles (including their portable document format (PDF) files) will be managed using EndNote X8.

#### Selection process

Pairs of authors will independently screen titles and abstracts for eligibility, followed by full text assessment. In the case of disagreement, a third reviewer will be consulted [47]. We will document the total numbers of retrieved references and the numbers of included and excluded studies in a flow chart, as recommended in the Preferred Reporting Items for Systematic Reviews and Meta-Analyses (PRISMA) statement [48].

#### Data collection process

In pairs, we will independently extract data according to a piloted form that will combine adapted versions of the Critical Appraisal and Data Extraction for Systematic Reviews of Prediction Modelling studies (CHARMS) checklist [38] to assess the methodological quality conduct of the included prognostic models and the Prediction Model Risk of Bias Assessment Tool (PROBAST) [36].

#### Data items

We will collect the following data about the selected studies and models:

- General information—author, title, source, publication date
- Source of data—for example, existing cohort, registry data
- Participants’ information—eligibility and recruitment method, study dates, treatments received, ethnicity, age and sex distributions
- Outcomes to be predicted—definition, blinding and time of measurement
- Candidate predictors—number, biomarkers included, and variables in the final model or model being validated. A list of potential biomarkers [see Additional file 2] that models might have considered will be included in the extraction form, with an option to record any additional HF-related biomarker encountered
- Information on missing data
- Model development—total sample size, total number of events, model name (if any), modelling method, assumptions assessment, predictors selection prior and during modelling, use of shrinkage techniques, testing for interactions, handling of continuous predictors
- Reporting of model—whether reported the final and other multivariable models including predictor weights, intercept, baseline survival (when appropriate), model performance measures (with standard errors or confidence intervals), and any alternative presentation of the final model
- Model validation—total sample size, total number of events, validation procedure (e.g. apparent, split-sample, other type of internal validation, external validation)
  - Internal validation—whether it was an apparent validation (i.e. without applying resampling techniques or hold-outs) or proper internal validation, i.e. using resampling methods (e.g., bootstrap or cross-validation) for building the model and not only for the final model. We will report if values have been adjusted for optimism.
  - External validation—target population, setting, data collection procedures. In cases of disappointing performance in external validation samples, we will report whether the model was updated in response, e.g. intercept recalibrated, predictor effects adjusted, or new predictors added. In cases of external validation, we will compare the list and distribution of predictors (that is, the mean and standard deviation, as well as the presence of missing data and/or missing predictors) for development and validation datasets, considering those of the development study as the reference.
- Model performance measures—calibration, discrimination, and overall performance measures. We will extract the corresponding estimates together with their standard error, 95% confidence interval, and (if applicable) *p* values, when reported and as appropriate. For calibration—the model’s ability to generate predicted probabilities similar to the observed probabilities—we will describe whether calibration plots, calibration slope, calibration intercept, Hosmer-Lemeshow goodness of fit test (for logistic models), and/or observed/expected outcomes ratio (O/E ratio) are reported. For discrimination—the model’s ability to correctly classify patients with and without the outcome of interest—we will report whether the area under the receiver operating characteristic (ROC) curve (AUC), concordance (c-index) statistic, D-statistic, and/or the log-rank test are presented. We will also report if other performance measures are presented, including *R*^2^ and the Brier score.

### Missing data

We will contact authors of individual studies for additional information, if required, particularly when there are missing performance measures and their variation estimates (i.e. standard deviation, and 95% confidence intervals). If such information does not become available, we will collect the following information instead, according to Debray et al.: [37]

- If no calibration measures are reported, we will extract information on: the mean predictor values (usually presented together with the sample characteristics); predicted number of events for the overall sample and/or predicted outcome probability and observed outcome probability (to be estimated from Kaplan-Meier curves in the presence of censoring); when available, observed and predicted outcomes across risk strata and/or observed and predicted outcome probabilities across risk strata.
- If no discrimination values are reported, we will extract information on: the distribution of the linear predictor (LP, i.e. linear combination of the model predictors in the study sample weighted by the regression coefficients of the model in the development study), i.e. overall variance of the LP; mean and standard deviation of the LP in individuals with the outcome; and mean and standard deviation of the LP in individuals without the outcome.

This information will allow us to estimate ln(O/E) and its variance and the logit(c) and its variance, quantities required for the meta-analysis of calibration and discrimination, respectively. These estimates will be obtained using the methods implemented in the R package metamisc [49]. If three or more studies are available and are clinically homogenous (e.g. similar prognostic factors, outcomes, prediction horizons, study conduct, purpose, quality), the same package will be used to meta-analyse model performance.

### Assessing risk of bias

The risk of bias in individual studies will be assessed using the Prediction Model Risk of Bias Assessment Tool (PROBAST) [36], which was developed to evaluate the extent to which shortcomings in the study design, conduct and analysis yield over- or under-estimated model predictive performance values. PROBAST also evaluates the applicability or extent to which the prognostic study assessed matches the systematic review research question in terms of population, predictors, and outcomes. PROBAST consists of 20 signalling questions grouped in four domains: participant selection; predictors; outcome; and analysis. The individual items of this tool will be embedded in the relevant sections of this review’s data-extraction form. An overall judgement will be made, reporting a ‘low’, ‘high’ or ‘unclear’ risk of bias and ‘low’, ‘high’, or ‘unclear’ concerns regarding applicability according to the tool guidelines.

### Publication bias

Unlike randomised control trial studies, prognostic modelling studies are typically not prospectively registered and usually no protocol is published [50]. Although difficult to estimate from reported data, we will evaluate and discuss the potential presence of publication bias.

### Data analysis and synthesis

For each HF prognostic model identified by our search strategy, we will tabulate the following information: participant population (specifying type of HF, setting and total sample size), model (name or brief description if no name available, type of statistical model, number of prognostic factors, biomarker(s) investigated, discrimination, calibration, internal validation method and presentation format of the model), and outcome (type, definition, prediction horizon and number of events).

For prognostic models that have been externally validated, an additional tabular display will be used to show: validation study identifier; participant population (specifying type of HF); setting; whether all prognostic factors in the original model were available and similarly measured in the external validation population; whether the original mathematical expression was used to estimate outcome probabilities; number of events/sample size; discrimination; calibration; any updates to the model.

This project plan consists of a number of systematic reviews. Hence, we will not pool all findings in one report but rather, we will focus on a subset of studies (models) where a summary and/or meta-analysis are feasible and informative. The hierarchy of decisions will start form HF types, go down to summarising derivation models grouped by clinical outcome reported, and finally carry out meta-analysis of performance estimates (extracted from external validation studies) of one model and one outcome (single or composite as per Table 1) at a time.

More specifically if sufficient data are available and if the corresponding studies have a fair degree of similarities in terms of their PICOTS, we will meta-analyse the predictive performance estimates of each model, provided that their risk of bias is negligible, using random effects models with weights given by the within-study error variance, to account for the expected amount of between-study heterogeneity. To obtain accurate summary estimates and to avoid excluding studies with poor reporting of performance measures, we will use multivariate meta-analysis [37]. If a particular model has been validated in three or more occasions, we will pool the results by applying meta-analyses and meta-regression. Meta-analyses will be performed using the *R* packages metamisc*,* and metafor (for meta-regression) [49].

As a sensitive search strategy will be used, we expect to observe a large amount of clinical as well as statistical and design heterogeneity amongst included studies. For each type of HF, we will explore the impact of the following design features known to affect the predictive performance of prognostic models for studies reporting models that contain similar predictors:

- Participants characteristics, including study dates to cover for improvements in biomarker measurement techniques, and study setting (e.g. primary or secondary care)
- Outcome definition, method and measurement time
- Number of candidate predictors, predictor selection methods, and handling of predictors
- Sample size and number of events
- Handling of missing data
- Type of reported predictive performance measures
- Differences between development and external validation populations

Overall between-study heterogeneity, particularly for performance measures of calibration and discrimination, will be assessed using the *I*^2^ statistic. Because this measure can be misleading, we will complement the assessment estimating Kendall’s tau and approximate 95% prediction intervals (which provide a range for the potential performance in a new validation study) will be calculated to further interpret the relevance of any between-study heterogeneity [50].

If ten or more studies are available, we will perform meta-regression analyses, where feasible, for biomarker(s); prediction horizon; setting; co-morbidities; studies assessing the performance of original models; studies assessing the performance of updated models (recalibrated or adjusted); studies assessing particular models.

Potential methodological influences will be explored using sensitivity analysis by temporarily removing from the analysis studies with high risk of bias for at least one domain of PROBAST. If study quality is low or if the between-study heterogeneity is high, we will report results as a narrative.

### Summary of findings

Currently, we are not able to assess the quality of the evidence using the GRADE (Grading of Recommendations, Assessment, Development and Evaluations) process, as GRADE guidance for prognostic models has not been developed yet. Instead, we will present in our summary of findings the biomarkers included in each model, the original and updated models, their predictive performance (apparent, internal, and external, if reported), population characteristics, most common predictor factors, and the clinical outcomes considered in this review that are listed in Table 1.

## Discussion

This project will consist of a number of systematic reviews that will allow us to assess the characteristics of prognostic models for HF which consider and/or include essential biomarkers, appraise their methodological conduct, and that of subsequent studies assessing the models’ predictive performance in populations other than the one used for the models’ development (referred to as external validation).

We envisage a very high yield of titles from the searches, from which only a small percentage will be eligible for inclusion. This is because the current recommended prognostic filters [33] include very broad criteria, hence the high yield. From a scoping search, we found that approximately 6% of the titles of an original search would be eligible for inclusion.

Additionally, it is anticipated that selecting the eligible papers may require training the not-statistically minded team members in prognostic modelling matters.

If sufficient data are available from the eligible studies, we will meta-analyse the models’ predictive performance. This evidence will guide future HF prognostic model design and contribute to improved HF clinical management.

Any important future protocol amendments as a result of insight acquired during the project development stages, will be documented in detail in a separate section titled ‘Differences from original protocol’ and justification for all changes will be offered.

## Supplementary information

**Additional file 1.** Heart failure prognostic biomarker models searches. This file contains the search strategies used to identify relevant studies in MEDLINE, EMBASE, and WoK

**Additional file 2.** List of potential biomarkers. This file contains a non-exhaustive list of possible HF-related biomarkers a prognostic HF model could include at the start of the model development process or retained in the final stage

**Additional file 3.** Preferred reporting items for systematic review and meta-analysis protocols (PRISMA-P) 2015

## Data Availability

Not applicable. This is only a protocol—no data were generated or used.

## Abbreviations

AHF: Acute heart failure
AUC: Area under the receiver operating characteristic (ROC) curve
BiVAD: Biventricular assist device
BNP: B-type natriuretic peptide
CHARMS: Critical Appraisal and Data Extraction for Systematic Reviews of Prediction Modelling studies
CHF: Chronic heart failure
CRT: Cardiac resynchronization therapy device
EMBASE: Excerpta Medica database
ECS: European Society of Cardiology
HF: Heart failure
hs-cTnT: High-sensitive cardiac troponin T
ICD: Implantable cardioverter-defibrillator device
LP: Linear predictor,
i.e: linear combination of the model predictors in the study sample weighted by the regression coefficients of the model in the development study
LVAD: Left ventricular assist device;
MACE: Major adverse cardiovascular events
MAGGIC: Meta-Analysis Global Group in Chronic Heart Failure
NHS: National Health Service
NICE: National Institute for Health and Care Excellence
NT-proBNP: *N*-terminal prohormone B-type natriuretic peptide
O/E ratio: Observed/expected outcomes ratio
OMT: Optimum medical therapy
PMG: Cochrane Prognosis Methods Group
PRISMA: Preferred Reporting Items for Systematic Reviews and Meta-Analyses statement
PROBAST: Prediction Model Risk of Bias Assessment Tool
PROSPERO: International Prospective Register of Systematic Reviews
SHFM: Seattle Heart Failure Model
soluble ST2 or sST2: Soluble suppression of tumorigenicity-2
UK: United Kingdom
US: United States
WoK: Web of Science Core Collection
