# Supplementary material for: UMBRELLA protocol: systematic reviews of multivariable biomarker prognostic models developed to predict clinical outcomes in patients with heart failure
Source: Diagn Progn Res. 2020 Aug 26;4:13. doi: 10.1186/s41512-020-00081-4 (PMC7448313; doi:10.1186/s41512-020-00081-4)
Supplement: Supplementary file 2 — Additional file 2. List of potential biomarkers. This file contains a non-exhaustive list of possible HF-related biomarkers a prognostic HF model could include at the start of the model development process or retained in the final stage [file 41512_2020_81_MOESM2_ESM.docx]

List of potential biomarkers a prognostic heart failure model could include at the start of the model development process or retained in the final stage

| Num | Name | Abbreviation |
| --- | --- | --- |
|  | Adiponectin |  |
|  | Adrenolutin |  |
|  | Adrenomedullin | ADM |
|  | Albuminuria |  |
|  | Aldosterone |  |
|  | Angiotensin II |  |
|  | Annexin | AS |
|  | Arginine vasopressin | AVP |
|  | Asymmetric dimethylarginine | ADMA |
|  | Bacteriuria |  |
|  | Beta1-adrenergic autoantibodies | Beta1-AAbs |
|  | Big endothelin-1 |  |
|  | Blood urea nitrogen | BUN |
|  | Brain-derived neurotrophic factor |  |
|  | B-type natriuretic peptide | BNP |
|  | Carbohydrate antigen 125 | CA125 |
|  | Cardiotrophin-1 | CT-1 |
|  | Catestatin | CST |
|  | Chromogranin A |  |
|  | Circulating amyloid-beta (1-40) |  |
|  | Circulating osteonectin |  |
|  | Circulating VE-catherin |  |
|  | Clusterin/apolipoprotein |  |
|  | Coenzyme Q10 | Q10 |
|  | Collagen propeptides |  |
|  | Combined free light chains | cFLCs |
|  | Copeptin |  |
|  | Copper | Cu |
|  | C-reactive protein | CRP |
|  | Creatinine |  |
|  | Creatinine excretion rate |  |
|  | Cystatin-C |  |
|  | D-dimers |  |
|  | Eicosatetraenoic acid |  |
|  | Endostatin |  |
|  | Endothelin-1 | ET-1 |
|  | Erythropoietin | EPO |
|  | Estimated glomerular filtration rate | eGFR |
|  | Fas |  |
|  | Ferritin |  |
|  | Fibroblast growth factor 23 | FGR-23 |
|  | Galectin-3 | Gal-3 |
|  | Glucose |  |
|  | Glycosylated haemoglobin | HbA1c |
|  | Gremlin-1 | Grem1 |
|  | Growth differentiation factor 15 | GDF-15 |
|  | Haemoglobin | Hg or Hb or Hgb |
|  | Hepatocyte growth factor | HGF |
|  | High-mobility-group box 1 | HMGB1 |
|  | Inorganic phosphate |  |
|  | Insulin-like Growth Factor Binding Protein 2 | IGFBP2 |
|  | Interleukins (e.g. IL-6, and others) | IL- |
|  | Leukocyturia |  |
|  | M2 muscarinic receptor autoantibodies | M2-AAbs |
|  | Matrix metalloproteinase | MMP |
|  | Membrane-bound ST2 | ST2 |
|  | Micrornas |  |
|  | Midregional pro-ADM | MR-proADM |
|  | Myeloperoxidase | MPO |
|  | Myostatin |  |
|  | N-acetyl-beta-D-glucosamidase | NAG |
|  | Neopterin |  |
|  | Neprilysin | sNEP |
|  | Neurohormone-cytokine level(s) (represents a group of biomarkers) |  |
|  | Neutrophil gelatinase-associated lipocalin | NGAL |
|  | Neutrophil to lymphocyte count | NLR |
|  | Norepinephrine |  |
|  | N-terminal probnp | NT-proBNP |
|  | Osteopontin |  |
|  | Osteoprotegerin |  |
|  | Oxidized low-density lipoproteins |  |
|  | Pentraxin 3 | PTX3 |
|  | Plasma fibroblast growth factor 23 | FGF23 |
|  | Plasma relaxin |  |
|  | Platelet to lymphocyte count | PLR |
|  | Potassium | K |
|  | Proenkephalin A | PENK |
|  | Red blood cell distribution width | RDW |
|  | Regulatory T cells | Tregs |
|  | Renin |  |
|  | Serum heart-type fatty acid-binding protein level | H-FABP |
|  | Serum uric acid |  |
|  | Sodium | Na |
|  | Soluble intercellular adhesion molecule-1 | sICAM-1 |
|  | Soluble receptor for advanced glycation end products | sRAGEs |
|  | Soluble ST2 | sST2 |
|  | Stromal cell-derived factor 1 |  |
|  | Surfactant protein type B | SP-B |
|  | Symmetric dimethylarginine | SDMA |
|  | Syndecan-1 |  |
|  | Syndecan-4 |  |
|  | Telomere length |  |
|  | Testosterone |  |
|  | Thyroid stimulating hormone | TSH |
|  | Tissue inhibitor of metalloproteinases | TIMP |
|  | Transferrin saturation |  |
|  | Transthyretin | TTR |
|  | Transtubular potassium concentration gradient | TTKG |
|  | Triiodothyronine | T3 |
|  | triiodothyronine Free | FT3 |
|  | Trimethylamine N-oxide | TMAO |
|  | Troponin I |  |
|  | Troponin T |  |
|  | Tumor necrosis factor α | TNF-α |
|  | Urinary [TIMP-2]x[IGFBP7] |  |
|  | Urinary C-type natriuretic peptide | CNP |
|  | Urinary NGAL |  |
|  | Urocortin-1 | UCN-1 |
|  | Vaccenic acid |  |
|  | Vitamin D |  |
